# *Rickettsia* disrupts and reduces endothelial tight junction protein zonula occludens-1 in association with inflammasome activation

**DOI:** 10.1128/iai.00468-24

**Published:** 2024-12-16

**Authors:** Loka Reddy Velatooru, Esteban Arroyave, Meagan D. Rippee-Brooks, Megan Burch, Ethan Yang, Bing Zhu, David H. Walker, Yang Zhang, Rong Fang

**Affiliations:** 1 Department of Pathology, The University of Texas Medical Branch 12338 , Galveston, Texas, USA; 2 Center for Biodefense and Emerging Infectious Diseases, The University of Texas Medical Branch 12338 , Galveston, Texas, USA; 3 Institute for Human Infections and Immunity, The University of Texas Medical Branch 12338 , Galveston, Texas, USA; 4 Department of Pharmacological and Pharmaceutical Sciences, College of Pharmacy, University of Houston 465312 , Houston, Texas, USA; Washington State University, Pullman, Washington, USA

**Keywords:** inflammasome, ZO-1, *Rickettsia*, endothelial cells, tight junction

## Abstract

*Rickettsia* spp. cause life-threatening diseases in humans. The fundamental pathophysiological changes in fatal rickettsial diseases are disrupted endothelial barrier and increased microvascular permeability. However, it remains largely unclear how rickettsiae induce microvascular endothelial injury. In the present study, we demonstrated that *Rickettsia conorii* infection disrupts the continuous immunofluorescence expression of the interendothelial tight junction protein, zonula occludens-1 (ZO-1), in infected monolayers of microvascular endothelial cells (MVECs), accompanied by significantly diminished total expression levels of ZO-1. Interestingly, *R. conorii* activated inflammasome in MVECs, as evidenced by cleaved caspase-1 and IL-1β in the cell lysates in association with significantly elevated expression levels of nucleotide binding and oligomerization domain, leucine-rich repeat, and pyrin containing protein 3 (NLRP3). Furthermore, selective inhibition of NLRP3 by MCC950 significantly suppressed the activation and cleavage of caspase-1 induced by *R. conorii* in endothelial cells, which further prevented the disruption of interendothelial junctions and reduction of ZO-1 expression. Of note, pharmaceutical inhibition of NLRP3 mitigated the disrupted endothelial integrity caused by *R. conorii*, measured by fluorescein isothiocyanate-dextran passage in a Transwell assay, independent of bacterial growth and cellular cytotoxicity. Taken together, our results suggest that *R. conorii* affected microvascular endothelial junction integrity likely via diminishing and interrupting the junctional protein ZO-1 in association with activating NLRP3 inflammasome. These data not only highlight the potential of ZO-1 as a biomarker for *Rickettsia*-induced microvascular injury but also provide insight into targeting NLRP3 inflammasome/ZO-1 signaling as a potentially adjunctive therapeutic approach for severe rickettsioses.

## INTRODUCTION

Tick populations and tick-borne diseases are spreading worldwide (1, 2). Rickettsiae of the spotted fever group (SFG) are Gram-negative, obligately intracellular bacteria which are transmitted to humans by tick bite. Although tick-borne SFG rickettsioses are often underreported due to lack of a reliable acute diagnostic assay, more than 6,000 cases of SFG rickettsioses are reported in the USA annually (3). Rickettsiae cause potentially life-threatening infections in otherwise healthy individuals with a reported case fatality as high as 5%–10% in the USA (4–6) and as high as 30%–50% in Mexico (7, 8). Due to the significant morbidity and mortality, rickettsial diseases continue to pose a serious public health threat globally. In the early 1900s, the investigative pathologist S. Burt Wolbach examined human tissues from postmortem specimens of patients who had died of rickettsial disease (9–11). For the first time, Wolbach unambiguously showed the invasion of *Rickettsia rickettsii* and *Rickettsia prowazekii* into microvascular endothelial cells (MVECs) and demonstrated that fatal rickettsioses are fundamentally a vasculitis. Wolbach described vascular changes as endothelial swelling and extensive perivascular accumulation of mononuclear inflammatory cells (9–11). However, although decades of effort have been attempted, the pathogenic mechanisms underlying increased microvascular permeability in fatal rickettsioses remain to be determined.

Endothelial cells are the inner monolayer of blood and lymphatic vessels. The integrity and barrier function of endothelium are vital to maintain the tissue-fluid homeostasis by restricting the transport of proteins across the endothelial barrier in a size-selective manner (12). Endothelial cells comprise a monolayer of cells connected to each other by a complex set of junctional proteins. Interendothelial junctions connect endothelial cells to form a contiguous monolayer, which plays a key role in regulating vascular barrier function. Interendothelial junctions are composed of protein complexes of adherens junctions (AJs), tight junctions (TJs), and gap junctions (12). The proteins of the zonula occludens, including zonula occludens-1 (ZO-1), ZO-2, and ZO-3, are tight junction-associated scaffold proteins that bind to transmembrane proteins of tight junctions and the underlying cytoskeleton. ZO-1 is a member of the membrane-associated guanylate kinase homolog family, which is a critical regulator of TJ assembly (13). Decreased expression of ZO-1 is associated with severe plasma leakage in various disease models (13–15).

Inflammasomes are cytosolic multiprotein complexes that recognize both pathogenic and danger stimuli in host cells, triggering the inflammatory response as part of the innate immune system. The NLRP3 inflammasome is the best characterized member of the nucleotide-binding and oligomerization domain-like receptor family, which consists of a sensor (NLRP3), an adaptor (apoptosis-associated speck-like protein containing a caspase activation recruitment domain, ASC), and an effector (caspase-1) (16). Activation of NLRP3 inflammasome leads to active enzymatic activity of caspase-1, which cleaves the precursors of inflammatory cytokines to active IL-1β, IL-18, and gasdermin D, further resulting in pyroptosis (16, 17).

We recently illustrated that *Rickettsia australis* activates NLRP3/ASC/caspase-1 inflammasome cascades in both mouse and human macrophages (18). Activation of inflammasome by rickettsiae in macrophages leads to secretion of biologically functional inflammatory cytokine IL-1β, which mediates rickettsicidal activity (18, 19). The experimental models of rickettsioses further elucidated that ASC, but not NLRP3 inflammasome, is essential for the host protective immune response against *R. australis* (18, 20). It has been increasingly recognized that aberrant inflammasome activation is critically involved in endothelial dysfunction in a variety of human diseases (21–23) but has not been investigated in severe infections caused by intracellular pathogens such as rickettsiae.

In this study, to investigate the molecular basis by which rickettsiae induce microvascular hyperpermeability, we studied the interactions of interendothelial junction with *R. conorii*, the etiologic agent of Mediterranean spotted fever. We sought to test the hypothesis that *Rickettsia* disrupts endothelial junctions via activating NLRP3 inflammasome. Among different endothelial junction proteins involved in maintaining the integrity of the endothelial cell layers, our studies focused on the tight junction protein ZO-1. We evaluated whether *R. conorii* interrupted endothelial integrity by altering the expression of ZO-1. In addition, we treated *Rickettsia*-infected endothelial cells with a selective NLRP3 inhibitor and observed its effect on endothelial cells, including the continuous expression of junction protein, NLRP3 inflammasome activation, rickettsial replication, and endothelial permeability. Our results highlight that *R. conorii*-induced NLRP3-associated reduction and disruption of ZO-1 likely lead to interrupted endothelial junction integrity that is the fundamental pathophysiological effect of severe rickettsioses.

## MATERIALS AND METHODS

### Rickettsia

*Rickettsia conorii* (Malish 7 strain) was obtained from the American Type Culture Collection (ATCC) (VR 613; Manassas, VA). *Rickettsia conorii* was cultivated in Vero cells, purified by a cushion of OptiPrep and resuspended in SPG (sucrose-phosphate-glutamate buffer [0.218-mmol/L sucrose, 3.8-mmol/L KH_2_PO_4_, 7.2-mmol/L K_2_HPO_4_, 4.9-mmol/L monosodium glutamic acid, pH 7.0]) as stock and stored at −80°C as described previously (18, 19). The concentration of rickettsiae propagated in cell culture was determined by quantitative real-time PCR or plaque assay as described previously (24, 25). All the experiments described in this study were conducted in a certified biosafety level 3 laboratory at the University of Texas Medical Branch.

### Mouse microvascular endothelial cells and rickettsial infection

Mouse MVEC line EOMA was purchased from ATCC (CRL-2586; Manassas, VA). MVECs were cultured as described previously (22). In brief, MVECs were cultured in Dulbecco’s modified Eagle’s medium (Gibco, USA) containing 10% fetal bovine serum in a humidified incubator at 37°C with 5% CO_2_. Cells were passaged by trypsinization (Gibco, 25200–056) when they reached a confluent monolayer.

MVECs were infected with *R. conorii* at a multiplicity of infection (MOI) as indicated. To facilitate internalization, rickettsiae were added to cells in a low volume of medium, for example, 500 µL/well of a six-well cell culture plate or 200 µL/well of a 24-well cell culture plate. Cells were initially incubated at 34°C with 5% CO_2_ for 2 h, followed by incubation at 37°C with 5% CO_2_, and then collected at the indicated time points. Uninfected cells served as a negative control.

### Pharmaceutical inhibition of NLRP3 inflammasome

To inhibit the activation of NLRP3 inflammasome, uninfected and infected mouse endothelial cells were treated with MCC950 (AG-CR1-3615; AdipoGen Life Sciences, San Diego). MCC950 was prepared and added to the cells 3–5 h prior to infection at a concentration of 10 µM during incubation until cells were collected for further analysis. Cells treated with dimethyl sulfoxide (DMSO) (Thermo Fisher Scientific, catalog # D12345) served as negative controls.

### Immunofluorescence microscopic analysis of junction protein ZO-1 in MVECs

Immunofluorescence analysis was performed to evaluate the continuous expression of junction protein ZO-1 in MVEC monolayers as described previously (22). In brief, 1 × 10^5^ MVEC/mL were seeded and cultured until confluent on poly-l-lysine coated glass coverslips (neuVitro, GG-12-Pre) in a 24-well plate. Cells were infected with *R. conorii* at the indicated MOIs. At the indicated time points, cells were fixed with methanol:acetone (1:1), and then washed with phosphate buffered saline (PBS) followed by blocking with 3% bovine serum albumin in PBS for 30 min. For ZO-1 staining, the fixed cells were incubated first with a rabbit polyclonal antibody against ZO-1 (Thermo Fisher Scientific, 40–2200) followed by incubation with Alexa Fluor plus 594-conjugated secondary antibody (Thermo Fisher Scientific, A32740). In parallel, another set of infected cells was incubated with rabbit polyclonal antibodies directed against *Rickettsia massiliae*, which are antigenically cross-reactive and closely related with *R. conorii*. Alexa Fluor plus 488-conjugated goat anti-rabbit antibodies (Thermo Fisher Scientific, A32731) served as the secondary antibodies. Samples were mounted in prolong Drop-n-Stain EverBrite mounting medium with or without 4′,6-diamidino-2-phenylindole (DAPI, Biotin 23,008-T, or SouthernBiotech, 0100–20). Images were acquired and analyzed by confocal microscopy (New Zeiss LSM 880) or Echo Revolve microscopy. The mean fluorescence intensity of ZO-1 in confocal images of mock and *R. conorii*-infected MVECs was quantified by ImageJ.

Tight junctions were represented by histograms of ZO-1 fluorescent intensity. The relative intensity of ZO-1 was calculated by crossing a line at random cell-to-cell contact sites. Image noise was determined by averaging the cytoplasmic intensity in multiple random regions and subtracting this value from the ZO-1 fluorescence intensity.

### Western blot

To evaluate whether rickettsiae activate inflammasomes in endothelial cells, uninfected and infected MVECs were collected and lysed in Cell Lysis Buffer (Cell Signaling, 9803) with phenylmethanesulfonyl fluoride (Cell Signaling, 8553) to inhibit serine proteases. After sonication, total protein was extracted and quantified by BCA Assay (Thermo Fisher Scientific, 23227). Extracted proteins (20–25 µg/well) were separated by sodium dodecyl-sulfate polyacrylamide gel electrophoresis and then transferred to polyvinylidene difluoride membrane (Thermo Fisher Scientific, 88518). After blocking, membranes were probed by antibodies specific for NLRP3 (Cell Signaling, D4D8T, 15101), caspase-1 (Cell Signaling, 2225), IL-1β (Cell Signaling, 12507), and ZO-1 (Thermo Fisher Scientific, 40–2200), individually, followed by horseradish peroxidase-conjugated appropriate secondary antibodies. Glyceraldehyde-3-phosphate dehydrogenase (Cell Signaling, D16H11, 5174) served as loading controls. The membrane was developed using Pierce Enhanced Chemiluminescence Western Blotting Substrate (Thermo Fisher Scientific, 32106). Densitometry analysis was performed using ImageJ software as described previously (19).

### Quantification of bacterial loads by quantitative real-time PCR

To determine the concentrations of intracellular rickettsiae following *in vitro* MVEC infection, *R. conorii*-infected MVECs were collected at 48 h post-infection (p.i.), and DNA was extracted from these cells using DNA extraction kit (Qiagen, Valencia, CA, USA) as described previously (19, 20, 24). Quantitative real-time PCR was performed using the iCycler from Bio-Rad (Hercules, CA, USA). Concentrations of *R. conorii* in endothelial cells were determined by real-time PCR with primers and TaqMan probes for the *Rickettsia*-specific citrate synthase (CS) gene (*gltA*) as described in our previous studies: *gltA* forward, GAGAGAAAATTATATCCAAATGTTGAT; *gltA* reverse, AGGGTCTTCGTGCATTTCTT; and *gltA* probe, CATTGTGCCATCCAGCCTACGGT. The *gltA* probe was labeled with 6-carboxyfluorescein (FAM) and Black Hole Quencher 1 (Biosearch Technologies, Petaluma, CA, USA). Two-step cycle parameters (95°C and 60°C) were used. The results were normalized to the amount of genomic DNA in the same sample and expressed as CS copy number per microgram of genomic DNA.

### Lactate dehydrogenase (LDH) cytotoxicity assay

A CytoTox96 non-radioactive cytotoxicity assay (Promega, G1780) was used to measure percent cytotoxicity according to the manufacturer’s instructions as described previously (13). In brief, supernatants of MVECs infected with *R. conorii* and treated with or without MCC950 were collected at 48 h p.i. Cells treated with TritonX-100 (Amresco, 0694) served as controls. After incubation with assay buffer and assay substrate, the absorbances of the wells in plates were read at 490 nm using the SpectraMax iD5 microplate reader (Molecular Devices, Sunnyvale, CA, USA). Cell death was calculated as the ratio of the LDH activity in the supernatant to the maximum LDH activity of lysed cells.

### Measurement of microvascular endothelial cell permeability

Endothelial permeability assays were performed with MVECs prepared on Transwell membrane inserts (0.4-µm pore size, Falcon Cell Culture Inserts; Thermo Fisher Scientific, catalog # 353095). MVECs were seeded at a density of 5 × 10^4^ cells in the upper compartment of each Transwell apparatus in a 24-well culture plate. After cells grew to full confluent monolayer, cells were infected and/or treated as indicated, and the endothelial permeability was examined as described previously with minor modifications (26, 27). Briefly, fluorescein-isothiocyanate (FITC)-dextran (average molecular weight 40,000; Sigma-Aldrich, St. Louis, MO, USA; catalog # 53379) was added to each upper well at a concentration of 100 µg/mL. After 30 min, permeability of FITC-dextran to the lower chamber was determined by measuring FITC fluorescence of the culture medium in the lower well with a fluorescence plate reader (SpectraMax iD5 microplate reader, Molecular Devices) using excitation at 485 nm and emission at 535-nm wavelength. MVECs exposed to DMSO vehicle or recombinant tumor necrosis factor (TNF)-alpha (200 ng/mL; Cell Signaling, 5178) served as negative and positive controls, respectively. The fluorescence intensity of the transferred FITC-dextran was obtained by subtracting the background value and expressed as fold change compared to normal control. The experiments were performed in triplicate.

### Statistical analysis

For comparison of multiple experimental groups, one-way analysis of variance with Bonferroni’s procedure was used. Two-group comparison was conducted using unpaired Student’s *t*-test. All the statistical analyses were performed using GraphPad Prism software, version 9.3. *P* values of 0.05 or less were the threshold for statistical significance.

## RESULTS

### *Rickettsia conorii* infection interrupted and diminished microvascular endothelial tight junctions

**Fig 1:**
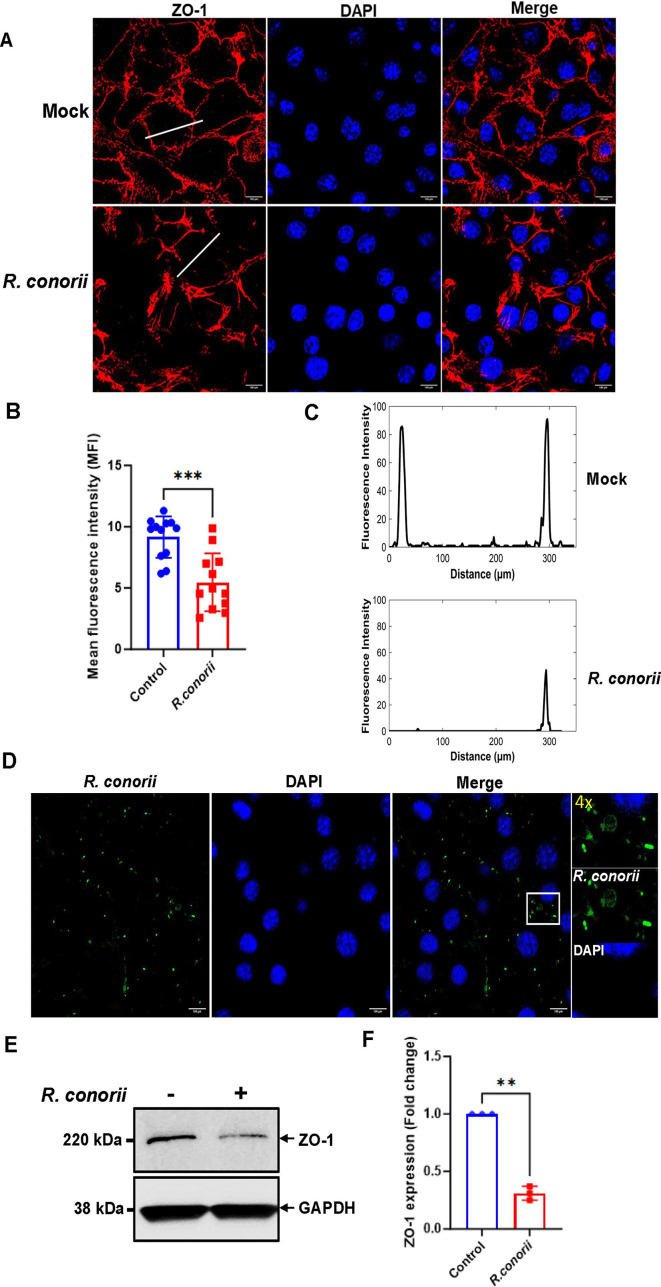
*Rickettsia conorii* induced discontinuous immunofluorescence staining of the junctional protein ZO-1 and diminished the expression levels of ZO-1 in cultured monolayers of microvascular endothelial cells. MVECs were cultured and plated as monolayers and then infected with *R. conorii* at an MOI of 2. Mock infected cells served as negative controls. At 48 h post-infection, the expression of endothelial tight junction protein ZO-1 (red) and nuclei (DAPI, blue) was determined by confocal immunofluorescence microscopy. The representative image obtained from the randomly selected field is shown (**A**). Quantification of the mean fluorescence intensity signal in confocal images of mock and *R. conorii*-infected MVECs is shown. The mean fluorescence intensity of the data from 12 randomly selected regions from three independent cultures is shown (**B**). Tight junctions are represented by histograms of ZO-1 fluorescence intensity as indicated by a white line across the two cell-cell contacts (**C**). *R. conorii* in infected MVECs was detected by confocal immunofluorescence microscopy. Rickettsiae are depicted in green, and nuclei (DAPI) are shown in blue (**D**). The expression levels of ZO-1 in the cell lysates were determined by immunoblotting using a specific antibody against ZO-1 (**E**). By densitometry analysis, the representative normalized ratios (fold changes) of ZO-1 to loading control GAPDH and to uninfected controls are shown (**F**). Representative results are shown from three independent experiments. Data represent mean ± SD, *n* = 3. **P < 0.01, ***P < 0.001. Scale bar = 100 µm. GAPDH, glyceraldehyde-3-phosphate dehydrogenase.

Endothelial cells are connected by tight junction proteins which maintain the integrity of the endothelium (12). Tight junctions function as a barrier in regulating paracellular permeability. ZO-1 is an essential tight junction protein which is associated with junction integrity, and its downregulation leads to junctional disruption and enhanced vascular permeability. To investigate the molecular basis of *Rickettsia*-induced microvascular hyperpermeability, we determined the expression of tight junction protein, ZO-1, in *R. conorii*-infected monolayers of MVECs at 48 h p.i. By using immunofluorescence confocal microscopy, we found that *R. conorii* induced discontinuous immunofluorescence expression of ZO-1 in the intercellular area of endothelial monolayers of MVECs (Fig. 1A). The immunofluorescence intensity of ZO-1 in *R. conorii*-infected MVECs was significantly reduced compared to uninfected controls (Fig. 1B). Through the white lines between cell-cell contacts as described previously (22, 28), we showed that ZO-1 was expressed in cell boundaries. Indeed, compared to uninfected controls, *R. conorii* infection interrupted the continuous immunofluorescence expression of ZO-1 (Fig. 1C). Additionally, the immunofluorescence intensity of ZO-1 across the cell-cell contacts of MVECs was dramatically reduced upon *R. conorii* infection (Fig. 1C and D). *R. conorii* significantly diminished the expression levels of the tight junction protein, ZO-1, in the total lysates of infected MVECs at 48 h p.i. (Fig. 1E and F). Our results showed significant discontinuous immunofluorescence expression of ZO-1 and disrupted interendothelial junctions accompanied by downregulated expression levels of ZO-1 in *R. conorii*-infected endothelial cells.

### Rickettsiae activated inflammasome in mouse endothelial cells

**Fig 2:**
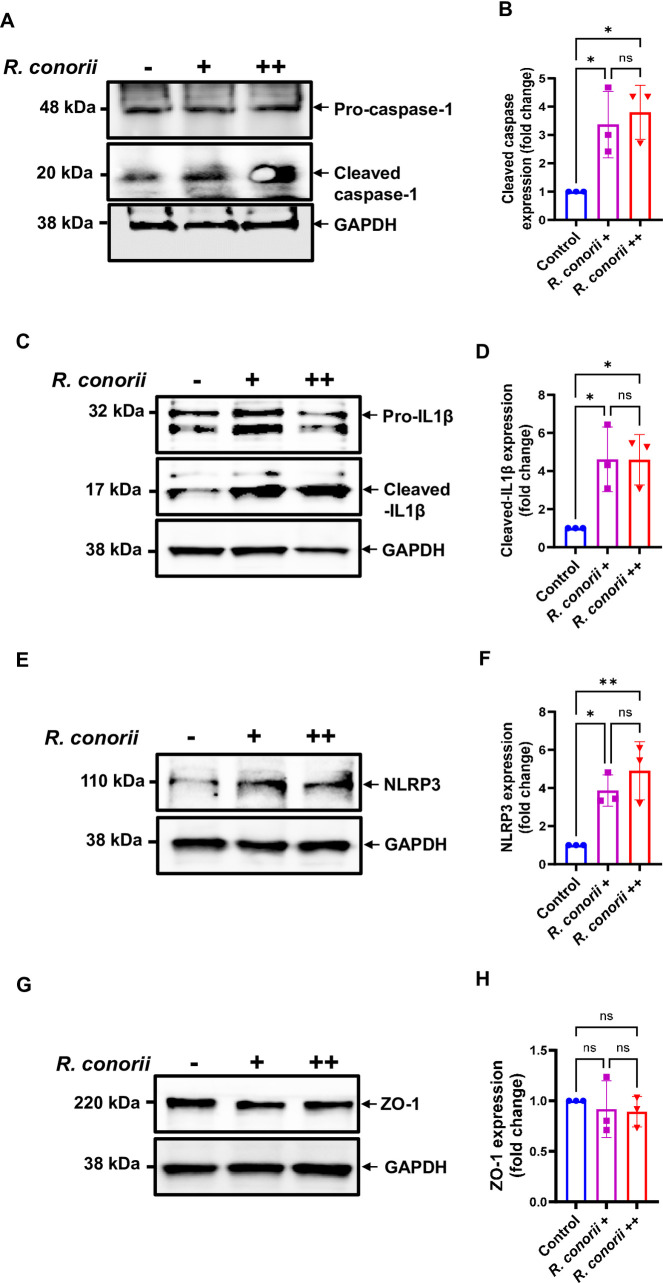
*Rickettsia conorii* activated inflammasome in association with upregulated expression levels of NLRP3 in endothelial cells. MVECs were infected with *R. conorii* at an MOI of 2 (+) or 10 (++). At 6 h p.i., cells were collected. Activation of inflammasome by *R. conorii* was determined by cleavage of pro-caspase-1 (**A and B**) and pro-IL-1β (**C and D**) into their activated forms by immunoblotting, respectively. The expression levels of NLRP3 (**E and F**) and ZO-1 (**G and H**) were determined using specific antibodies against NLRP3 and ZO-1, respectively. GAPDH served as loading control. The densitometry data show the representative fold changes as normalized ratios of activated caspase-1 (**B**), activated IL-1β (**D**), NLRP3 (**F**), and ZO-1 (**H**) to GAPDH and to controls. Data are expressed as means ± SD of three independent experiments. *P < 0.05, **P<0.01 (compared with the control group). ns, not statistically significant.

Increasing evidence has shown that NLRP3 inflammasome is a potential mediator of endothelial dysfunction (22, 23). To study the underlying mechanisms of *Rickettsia*-induced endothelial hyperpermeability, we first determined whether pathogenic rickettsial species, such as *R. conorii*, activate inflammasome in endothelial cells. As early as 6 h p.i., *R. conorii* triggered cleavage of pro-caspase-1 (p48) into activated caspase-1 p20 in the cell lysates of MVECs infected with both low and high doses of *R. conorii* (Fig. 2A and B). Furthermore, low and high doses of *R. conorii* also induced cleavage of pro-IL-1β into activated IL-1β in the cell lysates of infected MVECs (Fig. 2C and D). We found no significant differences in the expression levels of activated caspase-1 or IL-1β in cells infected with low versus high doses of *R. conorii*. These results suggest that *R. conorii* activates inflammasome in mouse microvascular endothelial cells.

To determine which NLR contributes to caspase-1-dependent inflammasome activation by *R. conorii* in endothelial cells, we examined the post-transcriptional expression levels of NLRP3 by immunoblotting. Compared to uninfected controls, endothelial cells infected with a low or high dose of *R. conorii* showed a significantly increased expression level of NLRP3 (Fig. 2E and F), suggesting that NLRP3 inflammasome contributes to cytosolic recognition of rickettsiae and leads to activation of caspase-1 and IL-1β in endothelial cells. Interestingly, no significant difference was observed in the expression levels of ZO-1 in *R. conorii*-infected MVECs at 6 h p.i. (Fig. 2G and H).

### Selective inhibition of NLRP3 blocked caspase-1 activation and preserved the post-transcriptional expression levels of interendothelial junctions interrupted by infection with *R. conorii*

**Fig 3:**
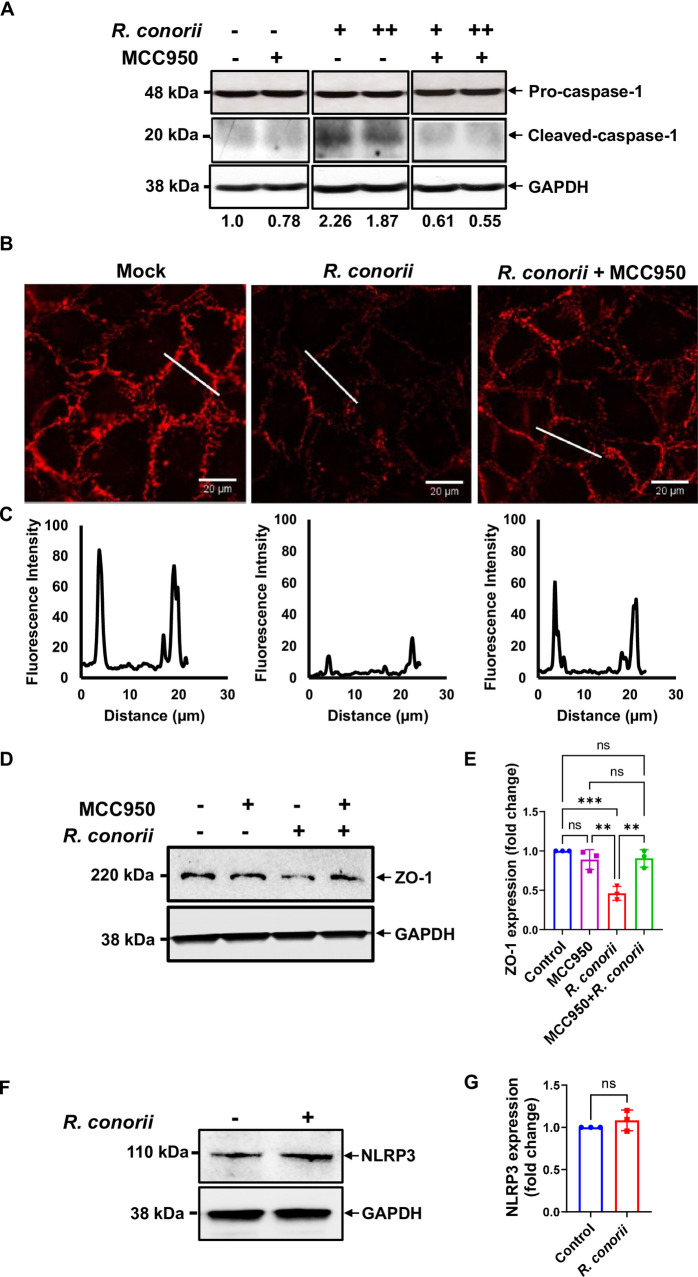
Selective inhibition of NLRP3 ameliorated the discontinuous immunostaining of ZO-1 and disrupted intercellular junctions caused by *R. conorii* infection in endothelial cell monolayers. MVECs were pre-treated for 3 h with MCC950 (10 µM) and infected with *R. conorii* at an MOI of 2 (+) or 10 (++). At 6 h p.i., cell lysates were collected, and the cleavage of pro-caspase-1 was determined by immunoblotting (**A**). At 48 h p.i., the continuous expression of endothelial tight junction protein ZO-1 in infected MVECs treated with or without MCC950 was determined by immunofluorescence microscopy (**B**). Tight junctions were represented by histograms of ZO-1 fluorescence intensity as indicated by a white line across the two cell-cell contacts (**C**). The expression levels of ZO-1 (**D and E**) and NLRP3 (**F and G**) at 48 h p.i. were determined by immunoblotting. The densitometry data show the representative fold changes as normalized ratios of ZO-1 (**E**) and NLRP3 (**G**) to GAPDH and to controls. Data are expressed as means ± SD of three independent experiments. **P<0.01, ***P<0.001 (compared with the control group). Scale bar = 20 µm. ns, not statistically significant.

To investigate whether NLRP3 inflammasome is associated with the activation of caspase-1 and endothelial dysfunction in rickettsial infection, we determined the effect of the selective NLRP3 inhibitor MCC950 on the expression levels of active caspase-1. As a pharmaceutical inhibitor specific against NLRP3, MCC950 does not inhibit the activation of other inflammasomes such as AIM2, NLRC4, or NLRP1 (29, 30). Interestingly, MCC950 treatment significantly reduced the levels of cleaved caspase-1 in MVECs infected with both low and high doses of *R. conorii* compared to untreated controls (Fig. 3A). Our results suggest that NLRP3 is associated with *R. conorii*-induced inflammasome activation in MVECs as evidenced by the observation that the selective inhibition of NLRP3 reduced the activation levels of caspase-1. These data also demonstrated that MCC950 effectively inhibited the activation of NLPR3 inflammasome by *R. conorii* in endothelial cells. Next, we determined the changes in the tight junction protein, ZO-1, in response to treatment with MCC950. Strikingly, MCC950 effectively reversed the impact of *R. conorii* infection on the disruption and reduction of interendothelial junctions, as demonstrated by the corrected continuous immunofluorescence expression of ZO-1 (Fig. 3B), reduced disruption, and increased fluorescence intensity of ZO-1 across the cell-cell contacts (Fig. 3B and C). Treatment with MCC950 reversed the diminished expression levels of ZO-1 caused by infection with *R. conorii*, which was close to those in uninfected cells (Fig. 3D and E). Interestingly, no significant changes were observed in the expression levels of NLRP3 in *R. conorii*-infected MVECs at 48 h p.i. (Fig. 3F and G). Therefore, these findings suggest that *R. conorii* interrupts endothelial junctions in association with inflammasome activation in the early stage of infection, most likely attributed to the NLRP3 inflammasome.

### Selective inhibition of NLRP3 inflammasome mitigated the endothelial barrier function disrupted by *R. conorii*

**Fig 4:**
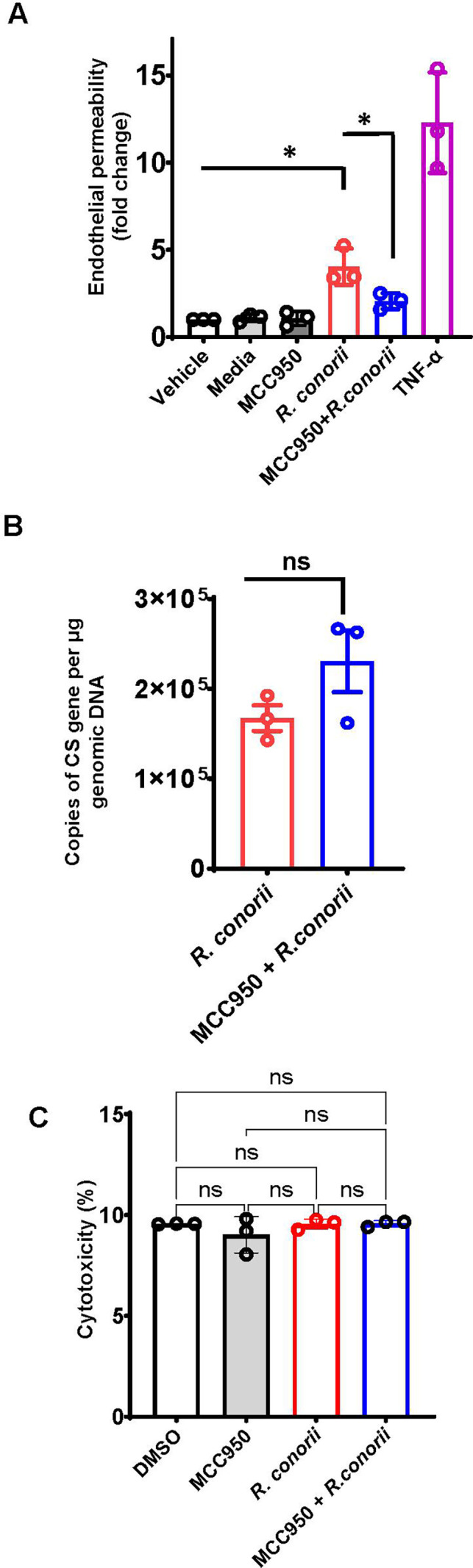
MCC950 mitigated the integrity of endothelial barrier function interrupted by *R. conorii* infection. MVECs were cultured in Transwell chambers and infected with *R. conorii*, with or without MCC950 (10 µM). The passage of 40-kDa FITC-dextran was quantified through the endothelial monolayer after 48 h. MVECs treated with DMSO vehicle, medium, or MCC950 served as negative controls. Recombinant TNF-alpha (200 ng/mL)-stimulated MVECs served as positive controls (**A**). At 48 h p.i., the concentrations of *R. conorii* in MVECs treated with MCC950 were determined by quantitative real-time PCR amplifying citrate synthase gene normalized to the amount of genomic DNA (**B**). The cytotoxicity of *R. conorii*-infected MVECs treated with or without MCC950 at 48 h p.i. was determined by LDH assay (**C**). Values represent mean ± SD of three replicates. *P < 0.05. ns, not statistically significant.

To further determine the contribution of inflammasome to increased microvascular endothelial permeability induced by rickettsial infection, MVEC monolayers were infected with *R. conorii* along with the NLRP3 inhibitor, MCC950. The permeability of endothelial cells was determined by passage of a FITC-dextran tracer using a Transwell system. The permeability of MVEC monolayers was increased three-fold by infection with *R. conorii* compared to uninfected controls and was significantly reduced by treatment with MCC950 (Fig. 4A). As a positive control, TNF-alpha-treated MVECs showed a dramatic increase in endothelial permeability (Fig. 4A). Next, we determined whether MCC950 mitigated the interruption and reduction of ZO-1 expression involving a mechanism of controlling rickettsial infection. Interestingly, inhibition of NLRP3 inflammasome activation did not significantly alter the concentrations of *R. conorii* in endothelial cells at 48 h p.i. (Fig. 4B), suggesting that MCC950 reversed the interrupted microvascular junctions independent of eliminating *R. conorii* in endothelial cells. At last, to exclude the possibility that *R. conorii* infection disrupts endothelial junctions via cell death, we determined the cytotoxicity by LDH assay. *R. conorii* infection did not induce significant cytotoxicity at 48 h p.i., as evidenced by the unaltered LDH level compared to uninfected cells (Fig. 4C). Therefore, these results demonstrated that pharmaceutical inhibition of NLRP3 inflammasome significantly ameliorated the *R. conorii*-induced disruption of endothelial barrier function via mechanisms independent of rescuing the survival of endothelial cells or reducing rickettsial concentrations.

## DISCUSSION

**Fig 5:**
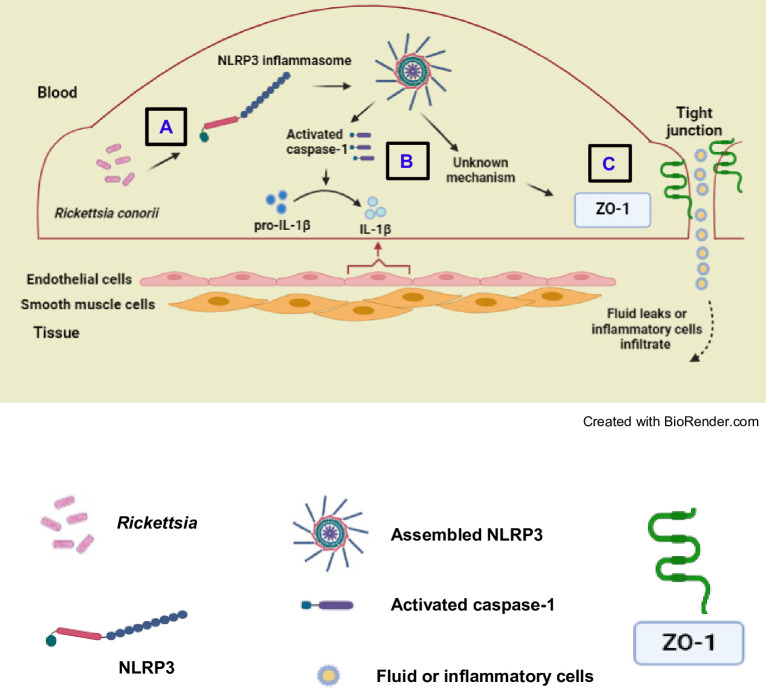
Schematic diagram of the proposed model of disruption of interendothelial tight junction ZO-1 by *R. conorii. Rickettsia conorii* invades microvascular endothelial cells and replicates in their cytoplasm. These obligately intracellular bacteria trigger activation of NLRP3 inflammasome (**A**). Assembly of NLRP3 multiprotein complex activates caspase-1, which induces cleavage of pro-IL-1β into IL-1β (**B**). Activation of NLRP3 inflammasome subsequently reduces and interrupts the cytoplasmic tight junction protein ZO-1 (**C**). These signaling pathways may open the tight junction complex, potentially increasing microvascular hyperpermeability, which results in transmigration of plasma fluid and/or infiltration of inflammatory cells from blood into peripheral tissues.

In this study, we demonstrated that *R. conorii* diminished and disrupted the expression of endothelial tight junction protein ZO-1 and interrupted the tight junction integrity of infected microvascular endothelial cell monolayers. We found that *R. conorii* activated caspase-1-dependent inflammasome, most likely NLRP3, in microvascular endothelial cells. Upon endocytosis, rickettsiae quickly escape endosomes and remain free in the cytoplasm of endothelial cells, where these Gram-negative bacteria further activate NLRP3 inflammasomes (Fig. 5A). Activation of NLRP3 inflammasome by *R. conorii* in endothelial cells leads to cleavage of pro-caspase-1 and pro-IL-1β into their biologically active forms (Fig. 5B). Our data presented here supported our hypothesis that *Rickettsia* activated NLRP3 inflammasome to induce the disruption of tight junction protein ZO-1 in microvascular endothelial cells in a mechanism independent of rickettsial clearance or cytotoxicity. The interrupted interendothelial junctions may lead to endothelial dysfunction and microvascular hyperpermeability, which prompts the leakage of fluid and infiltration of inflammatory cells from blood into perivascular tissues (Fig. 5C). Taken together, these results highlight the contributions of NLRP3 inflammasome and tight junction ZO-1 to endothelial pathophysiology in infectious diseases caused by tick-borne cytosolic-replicating bacteria.

We and others have recently reported that *Rickettsia* spp. activate the key element of the host cytosolic surveillance system, inflammasome (18–20, 31, 32). *Rickettsia parkeri* exploits caspase-11-dependent non-canonical inflammasome to counteract the anti-rickettsial effect mediated by type I interferon (IFN) (31). Most recently, caspase-1- and caspase-11-dependent IL-1 signaling was revealed to account for the difference in host-rickettsiae interactions in pathogenic versus non-pathogenic rickettsial species (32). Virulent *R. rickettsii* and *Rickettsia typhi* inhibit caspase-11- gasdermin D-IL-1α signaling to benefit their infections (32). *In vivo* host control of *R. australis* can be attributed to ASC-dependent inflammasome-mediated production of IFN-γ, which is essential for host defense against rickettsiae (20). Although NLRP3 contributes to the activation of ASC inflammasome by *R. australis* in macrophages, NLRP3 plays a negligible role in host protection *in vivo* (18), suggesting that NLRP3 is possibly involved in mediating pathogenesis of rickettsioses. These aforementioned studies have mostly focused on the interactions of *Rickettsia* with cytosolic recognition receptor inflammasome in macrophages, particularly in the aspect of host control of bacterial infection. Our studies demonstrated the activation of NLRP3 inflammasome by *R. conorii* in microvascular endothelial cells, which are the primary targets of highly virulent rickettsiae, and their contribution to the interrupted integrity of the endothelial barrier. More interestingly, pharmaceutical inhibition of NLRP3 inflammasome did not lead to any significant difference in the concentration of *R. conorii* in endothelial cells (Fig. 4). These results are distinct from the findings in previous studies, most of which have investigated how canonical or non-canonical inflammasome/inflammasome signal elements significantly benefit or eradicate rickettsiae in macrophages (18–20, 31, 32). It has been proposed that the pathogenic mechanisms of fatal rickettsioses mostly result from the overwhelming/uncontrolled replication of these obligately intracellular bacteria in endothelium. An earlier study reported that heparin prolongs the life of *in vitro R. rickettsii*-infected endothelial cells in a mechanism independent of the bacterial burden as a putative antioxidant (33). Our findings revealed the contribution of NLRP3 inflammasome, a rickettsial replication-independent cytosolic recognition signaling pathway, to rickettsiae-induced endothelial pathophysiology. Our work highlights the concept that rickettsiae trigger endothelial pathophysiological changes by both rickettsial replication-dependent and -independent mechanisms. It would be interesting in the future to determine how rickettsiae activate NLRP3 inflammasome in microvascular endothelial cells independent of bacterial replication.

*Rickettsia*-induced microvascular hyperpermeability and endothelial dysfunction constitute the fundamental pathologic changes in severe rickettsioses, such as Rocky Mountain spotted fever (RMSF). Endothelial injury and increased permeability also account for the typical clinical manifestations of rickettsioses including rash, meningoencephalitis, interstitial pneumonia, and non-cardiogenic pulmonary edema. Studies of the interactions of rickettsiae with microvascular endothelial cells have previously revealed several molecules and signaling pathways involved in *Rickettsia*-driven vascular inflammation and dysfunction. Earlier studies have demonstrated that *R. conorii* enhances the expression of adhesive molecules E-selectin, intercellular adhesion molecule-1, and vascular cell adhesion molecule-1, which potentially leads to vascular injury through promoting the adherence of mononuclear cells to endothelial cells (34). *In vitro* infection of endothelial cells with *R. conorii* or *R. rickettsii* promotes the upregulation of pro-inflammatory cytokines, such as IL-6 and IL-18 (35), and chemokines, such as CX3CL1 and MCP-1, at both transcriptional and post-transcriptional levels (36, 37). In addition, rickettsial infection triggers the activation of transcription factor NF-kappa B (38) and mechanistic target of rapamycin (39), and the regulation of enhancer long non-coding (elnc) RNAs NONMMUT024103/Apol10b (40), interferon-stimulated gene 15 (ISG15), and ISG15-specific protease UBP43 (41). Recently, *Rickettsia*-induced endothelial-derived exosomes have been reported to potentially disrupt the integrity of the human endothelial barrier (42). The microvascular hyperpermeability induced by *R. rickettsii* is associated with loss of interendothelial cell adhesion junctions, such as β-catenin and p120-catenin (p120), which are required to maintain vascular barrier function in conjunction with vascular endothelial (VE)-cadherin, the major adhesion molecules of adherens junctions in endothelial cells (43, 44). *R. montanensis* was found to phosphorylate VE-cadherin and directly attenuate endothelial adherens junctions (45). ZO-1 plays a crucial role in the assembly of functional TJs and AJs (46–48). ZO-1 also regulates the cross-interaction between TJs and AJs through control of intracellular tension and assembly of the VE-cadherin mechanosensory complex (49). Our findings provide the evidence that *R. conorii* not only reduced the expression levels of tight junction protein ZO-1, disrupting its continuous expression at the interendothelial cell junction, but also interrupted the endothelial barrier function. The cellular model of rickettsial diseases described here not only revealed a previously unappreciated molecule or biomarker for microvascular endothelial injury caused by rickettsiae, ZO-1, but also highlighted the potential contribution of ZO-1-dependent mechanisms of pathogenesis of rickettsioses. Reduction of ZO-1 has been shown to be associated with severe plasma leakage in patients and experimental models *in vivo* (14, 15, 49). Therefore, our findings suggest that *R. conorii* reduces microvascular junction integrity by activating NLRP3/ZO-1 signal cascades to promote the extravasation of plasma and leukocytes from blood vessels into tissues in severe rickettsioses.

As obligately intracellular bacteria, rickettsiae are known to modify or regulate host-cell death program for their benefit of survival or replication (37, 38). Highly virulent rickettsiae, such as *R. rickettsii*, suppress apoptotic machinery of infected endothelial cells at the early stage of infection through NF-kappa B-dependent mechanisms (38). The “Israeli spotted fever” strain of *R. conorii* induces increased endothelial permeability only at the late stage of infection as evidenced by diminished electrical resistance across the endothelial monolayer, which is accompanied by caspase-1-associated pyroptotic cell death (37). In line with these findings, our results demonstrated that *R. conorii* activated caspase-1 inflammasome accompanied by a significantly increased expression level of NLRP3 inflammasome, without significant effect on expression levels of ZO-1, at the very early stage of infection in mouse microvascular endothelial cells (Fig. 2). At the late stage of rickettsial infection in endothelial cells, *R. conorii* disrupted endothelial cell integrity, leading to reduced and discontinuous expression of ZO-1, while NLRP3 expression levels remained unaffected (Fig. 3 and 4). Furthermore, MCC950 specifically binds to both active and inactive NLRP3 via targeting the ATP hydrolysis motif, thereby blocking the ability of NLRP3 to hydrolyze ATP for NLRP3 inflammasome function (30). MCC950 has also been reported to suppress the cleavage of pro-capsase-1 into active p-20 fragment of caspase-1 (29), which is also supported by our findings in rickettsial infection. However, MCC950 has been recently reported to have potential off-target effects in macrophages, including targeting carbonic anhydrase 2 (CA2) (50). The inhibition of CA2 activity by MCC950 in endothelial cells has not been reported. Further investigations on the interactions of *R. conorii* with NLRP3 inflammasome in endothelial cells using genetic knockdown approaches such as siRNA or shRNA are warranted. In addition, a limitation of our research is that the findings on NLRP3 activation by *R. conorii* and the subsequent effect on endothelial junctions were derived from a mouse endothelial cell line. Although it remains unknown whether canonical, non-canonical, or alternative NLRP3 activation is involved, our findings revealed a previously unrecognized notion that NLRP3/caspase-1 accounts for a mechanism mediating the interrupted tight junction ZO-1 by *R. conorii* infection in endothelial cells.

In summary, our present study, for the first time, demonstrated the potential of ZO-1 as a biomarker for vascular dysfunction caused by highly virulent rickettsiae, such as *R. conorii*. This work not only identified the close association of interendothelial tight junction ZO-1 with interrupted microvascular barrier integrity but also revealed the indispensable role of NLRP3 inflammasome in driving ZO-1-mediated endothelial injury caused by rickettsial infection. These new findings may lead to a promising therapeutic strategy for severe rickettsioses by targeting NLRP3 to ameliorate endothelial permeability/inflammation/injury as an adjunctive approach to accompany anti-rickettsial treatment.
